# Post-induction hypotension and intraoperative hypotension as potential separate risk factors for the adverse outcome: a cohort study

**DOI:** 10.1007/s00540-023-03191-7

**Published:** 2023-04-21

**Authors:** Szymon Czajka, Zbigniew Putowski, Łukasz J. Krzych

**Affiliations:** 1 Department of Anaesthesiology and Intensive Care, School of Medicine in Katowice, Medical University of Silesia, 14 Medykow Street, 40-752 Katowice, Poland; 2 Students’ Scientific Society, Department of Anaesthesiology and Intensive Care, School of Medicine in Katowice, Medical University of Silesia, Katowice, Poland

**Keywords:** Arterial pressure, General surgery, Abdominal surgery, Hypotension, Post induction hypotension

## Abstract

**Purpose:**

Intraoperative hypotension (IOH) is associated with organ hypoperfusion. There are different underlying causes of IOH depending on the phase of surgery. Post-induction hypotension (PIH) and early-intraoperative hypotension tend to be frequently differentiated. We aimed to explore further different phases of IOH and verify whether they are differently associated with postoperative complications.

**Methods:**

Patients undergoing abdominal surgery between October 2018 and July 2019 in a university hospital were screened. Post-induction hypotension was defined as MAP ≤ 65 mmHg between the induction of anaesthesia and the onset of surgery. Hypotension during surgery (IOH) was defined as MAP ≤ 65 mmHg occurring between the onset of surgery and its completion. Acute kidney injury, stroke or transient ischaemic attack, delirium, and myocardial infarction were considered as the outcome.

**Results:**

We enrolled 508 patients (219 males, median age 62 years). 158 subjects (31.1%) met PIH, 171 (33.7%) met IOH criteria, and 67 (13.2%) patients experienced both. PIH time accounted for 22.8% of the total hypotension time and 29.7% of the IOH time. The IOH time accounted for 5.17% of the total intraoperative time, while PIH for 8.91% of the pre-incision time. Female sex, lower height, body mass and lower pre-induction BP (SBP and MAP) were found to be associated with the incidence of PIH. The negative outcome was observed in 38 (7.5%) patients. Intraoperative MAP ≤ 65 mmHg, longer duration of the procedure (≥ 230 min), chronic arterial hypertension and age were associated with the presence of the outcome (*p* < 0.01 each).

**Conclusions:**

The presence of IOH defined as MAP ≤ 65 mmHg is relevant to post-operative organ complications, the presence of PIH does not appear to be of such significance. Because cumulative duration of PIH and IOH differs significantly, especially in long-lasting procedures, direct comparison of the influence of PIH and IOH on outcome separately may be biased and should be taken into account in data interpretation. Further research is needed to deeply investigate this phenomenon.

## Introduction

Intraoperative hypotension (IOH) is a multifactorial phenomenon. Its occurrence is determined by disruption of either cardiac output (CO) or systemic vascular resistance (SVR) [1]. Disturbances within CO or SVR can be induced by a variety of patient- and procedure-related factors, including bleeding, drug-mediated vasodilation or cardiac depression [2]. Despite a substantial body of observational data, no universally accepted IOH definitions have been proposed [3]. Nevertheless, IOH, (as a whole,) is associated with organ hypoperfusion which manifests in perioperative organ dysfunction. Some of the most commonly reported complications are acute kidney injury (AKI), myocardial infarction (MI) and stroke [4].

As pointed out by Südfeld et al., there are different underlying causes of IOH depending on the phase of surgery. The authors have distinguished two different types of IOH, i.e. post-induction hypotension (PIH) and early-intraoperative hypotension [3]. However, it remains undetermined to what extent both of these phenomena can cause organ damage.

The purpose of this study is to explore further different phases of IOH and verify whether these phases are differently associated with postoperative complications.

## Materials and methods

The present study shares its data with a cohort study previously published by our team [5], in which we explored the role of intraoperative hypotension in patients with and without preoperatively diagnosed arterial hypertension. Patients who underwent abdominal surgery between October 1, 2018 and July 15, 2019 in a university hospital were screened. Organ retrieval procedures, re-operations, procedures performed under local anaesthesia and under monitored anaesthesia care, and those classified as immediate according to the NCEPOD Classification of Intervention were excluded [6]. Demographic and clinical data were recorded, including sex, age, weigh, height, comorbidities and their pharmacological treatment, according to the ICD 10 coding [7]. Body mass index (BMI) and Charlson Comorbidity Index (CCI) were subsequently calculated. The type and duration of anaesthesia as well as the type, duration and urgency of surgery were recorded. Perioperative risk was assessed based on individual patient’s risk, according to the American Society of Anesthesiology (ASA) physical status (PS) classification [8], and procedural risk, according to the European Society of Cardiology and European Society of Anaesthesiology recommendations [9]. Primary arterial hypertension was diagnosed based on medical records. The ongoing antihypertensive therapy was evaluated.

The study protocol included non-invasive measurement of systolic blood pressure (SBP), diastolic blood pressure (DBP): baseline (the first measurement in the operating theatre once monitoring has been initiated), before and after the induction of anaesthesia and at 5-min intervals until the patient was discharged from the operating theatre. Blood pressure was measured using an automatic oscillometric method (Dräger Infinity Gamma XL). The cuff size was adjusted to the arm circumference. The application of noradrenaline (NA) was analysed according to the dose and duration of infusion.

Post-induction hypotension was defined as MAP ≤ 65 mmHg occurring between the induction of anaesthesia and the onset of surgery. According to our hypothesis, in this phase, a factor clearly predisposing to hypotension is the effect of anaesthetics. The second type of hypotension analysed was hypotension during surgery (IOH), defined as MAP ≤ 65 mmHg occurring between the onset of surgery and its completion. In this phase of the surgical procedure, the factors related to surgical manoeuvres become more prominent.

Procedures were divided into two groups depending on the median duration of a procedure. In the postoperative period, the incidents of hypoperfusion of vital organs were recorded, and included the occurrence of acute kidney injury (AKI), stroke or transient ischaemic attack (TIA), delirium and myocardial infarction (MI), according to their international definitions [10–12]. This composite endpoint was considered the outcome.

All patients gave their written informed consent for data management. On the basis of the decision of the bioethics committee of the Medical University of Silesia in Katowice (no. PCN/CBN/0052/KB/116/22), the study was accepted, the committee confirmed that it was not necessary to obtain consent to conduct research involving the review of patient records. (Sect.  21 and 22 of the Act of 5 December 1996 on the Medical Profession in Poland).

The STROBE (STrengthening the Reporting of OBservational studies in Epidemiology) Statement was applied for appropriate reporting.

Statistical analysis was performed using MedCalc Statistical Software version 18.1 (MedCalc Software Ltd., Ostend, Belgium). Continuous variables were expressed as median and interquartile range (IQR). Qualitative variables were expressed as absolute values and/or percentages. Between-group differences for quantitative variables were assessed using the Mann–Whitney U-test or Kruskal–Wallis test. Their distribution was verified with the Shapiro–Wilk test. The chi-square test or Fisher’s exact test were applied for qualitative variables. Odds ratios (OR) with their 95% confidence intervals (CI) were calculated, if applicable. All tests were two-tailed. A p-value < 0.05 was considered statistically significant. Variables which reached p value of  < 0.1 in univariate analysis were included in a multivariable stepwise logistic regression model.

## Results

**Fig. 1:**
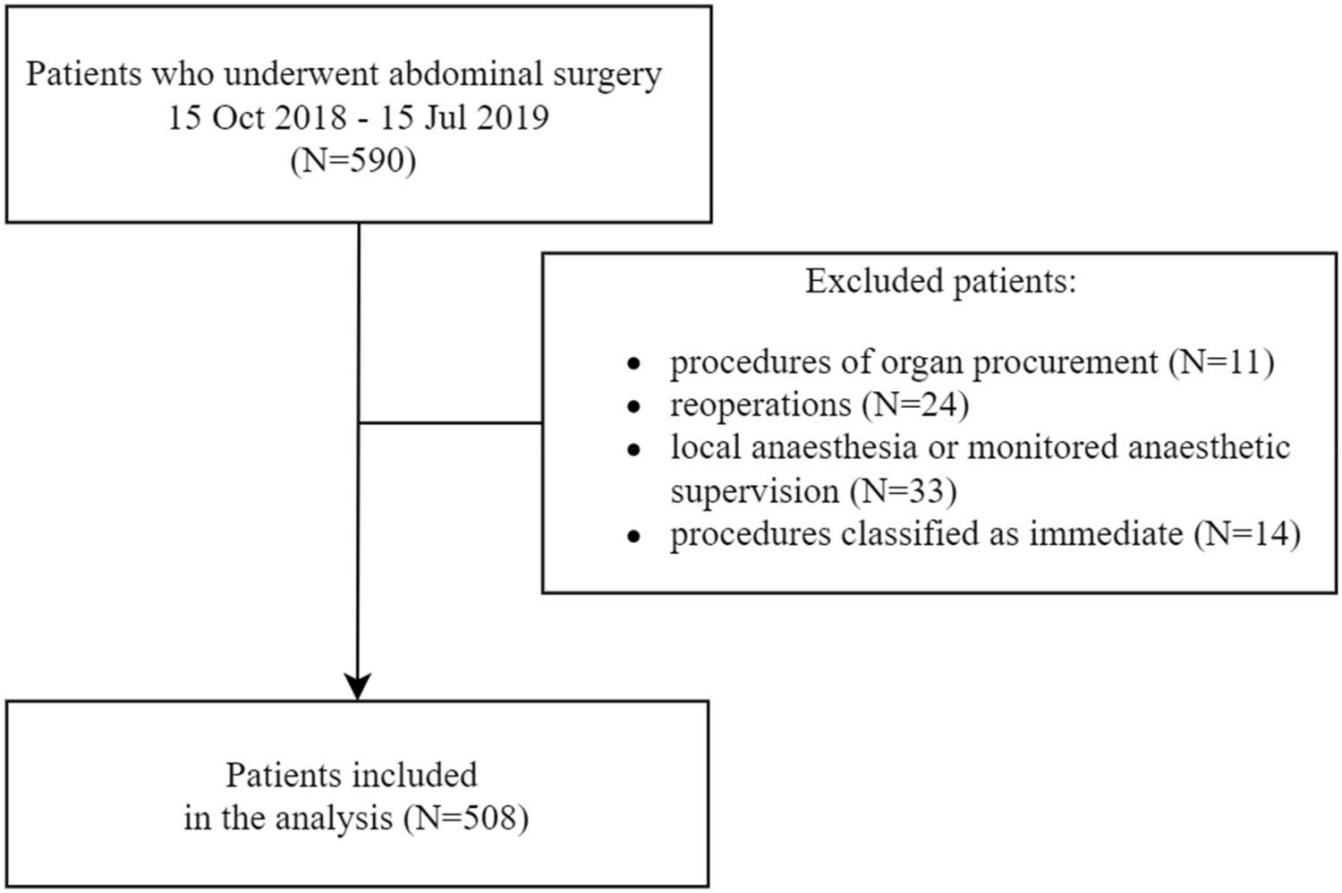
Flow diagram for the patient selection process

A total of 508 patients were included in the final analysis (Fig. 1). The study consisted of 219 (46.6%) males and 289 (53.4%) females. The median age of patients was 62 years (IQR 46–68). Arterial hypertension concerned 234 (46%) individuals preoperatively. Patients were treated with the following antihypertensive agents: beta-blockers (n = 133, 26.2%), angiotensin converting enzyme (ACE) inhibitors or angiotensin receptor blockers (ARB) (n = 110, 21.7%), calcium channel blockers (CCB) (n = 43, 8.5%), aldosterone receptor antagonists (n = 11, 2.2%), loop diuretics or thiazides (n = 73, 14.4%). Detailed study group characteristics are presented in Table 1. The median duration of anaesthesia was 230 min (IQR 130–340). Two groups were distinguished: group A—longer procedures lasting 230 min or more and group B—shorter procedures lasting less than 230 min.

**Table 1:** Anaesthesia- and surgery-related data

Variable		Value
Duration of anaesthesia (min)		230 (130–340)
Duration of surgery (min)		175 (90–280)
Type of anaesthesia	General	508 (100%)
	General + regional (any)	51 (10%)
ESC/ESA procedure-related risk	Low	45 (9%)
	Intermediate	335 (66%)
	High	128 (25%)
Urgency of procedure	Elective	459 (90%)
	Non-elective	49 (10%)
Type of surgery	Surgery of the pancreas	102 (20%)
	Surgery of the small intestine	74 (14.6%)
	Surgery of the large intestine	97 (19.1%)
	Cholecystectomy	113 (22.2%)
	Hernia repair surgery	56 (11%)
	Gastric surgery	25 (5%)
	Surgery of the oesophagus	8 (1.6%)
	Liver surgery	8 (1.6%)
	Splenectomy	3 (0.6%)
	Other abdominal surgery	22 (4.3%)
Oncological surgery		245 (48.2%)
Premedication (some patients received >1 drug)	Midazolam	250 (49.2%)
	Lorazepam	139 (27.4%)
	Hydroxizine	57 (10.2%)
	No premedication	204 (40.2%)

Qualitative variables are depicted as absolute value (and percentage); quantitative variables are shown as median (and interquartile range); ESA—European Society of Anaesthesiology, ESC—European Society of Cardiology

**Fig. 2:**
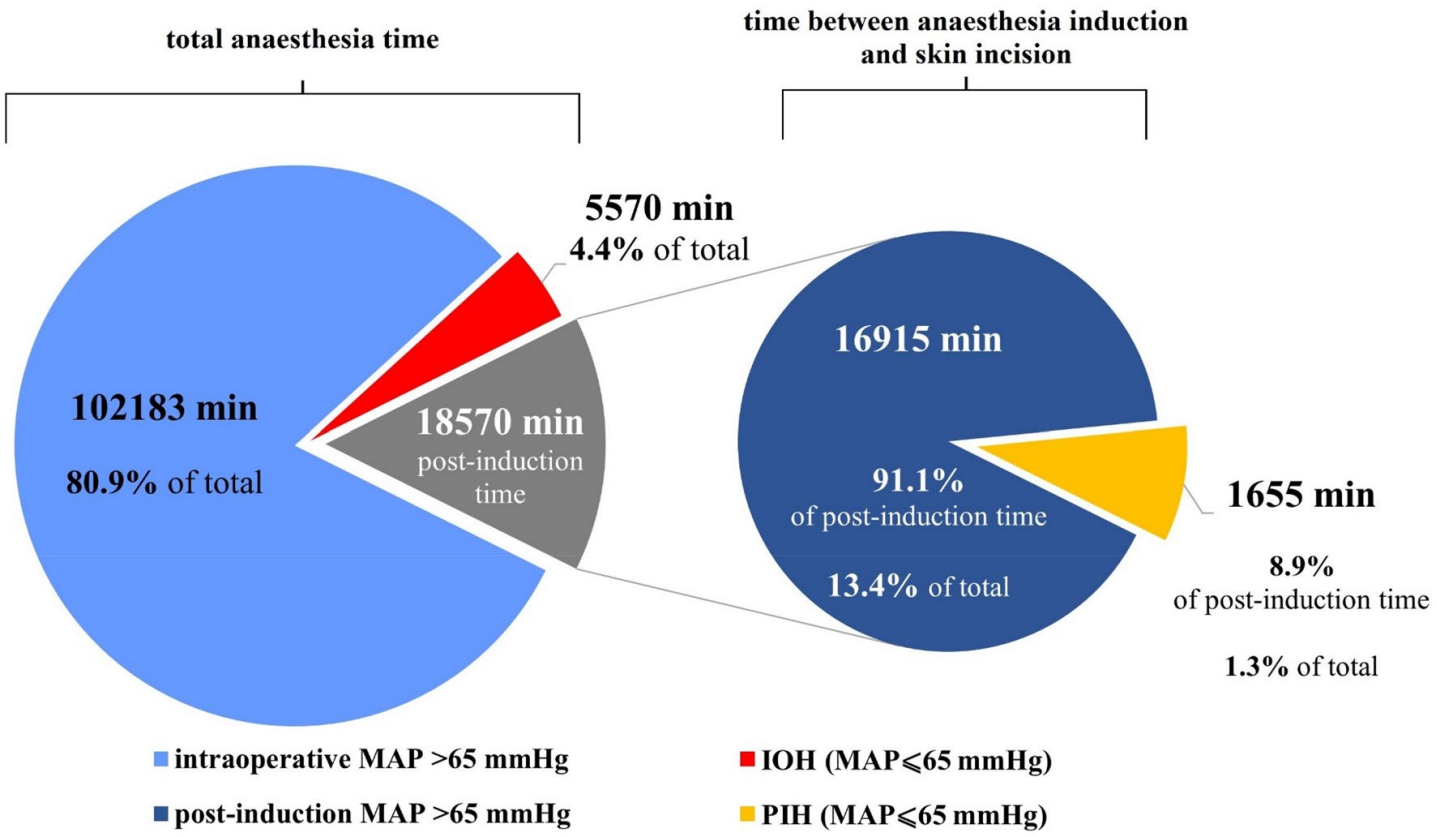
Cumulative time of occurrence of blood pressure values measured in the operating room divided according to the studied thresholds and periods of hypotension (min)

There were 49 (9.6%) non-elective procedures and 245 (48.2%) oncologic surgeries. All patients underwent general anaesthesia, supplementary regional techniques (i.e. neuraxial or peripheral blocks) were used in 51 (10%) of cases. The majority of patients 335 (66%) were at intermediate ESA/ESC procedure-related risk. The median pre-induction SBP was 140 mmHg (125–155), whereas median pre-induction MAP reached 101.7 (92.3–110.0) mmHg. Moreover, 158 subjects (31.1%) met PIH and 171 (33.7%) met IOH criteria, and 62 (12.2%) patients experienced both PIH and IOH. The recorded PIH time accounted for only 22.8% of the total hypotension time and 29.7% of the IOH time. The IOH time accounted for 5.17% of the total intraoperative time, while the PIH time accounted for 8.91% of the cumulative time between anaesthesia induction and skin incision. The cumulative hypotension times of each type are presented in Fig. 2. Female sex, lower height, body mass and lower pre-induction BP (SBP and MAP) were found to be associated with the incidence of PIH. In ASA I patients, the incidence of PIH was significantly lower and all 3 patients classified as ASA class V met PIH criteria (Table 2). On the other hand, the occurrence of IOH was found to be associated with 5 factors (Table 2).

**Table 2:** Variables related to the presence of PIH and IOH

Variable	All n = 508 (100.0)	Post-induction MAP ≤ 65 mmHg		P-value (PIH)	Intraoperative MAP ≤ 65 mmHg		P-value (IOH)
		Present n = 158 (31.1)	Absent n = 350 (68.9)		Present n = 171 (33.7)	Absent n = 337 (66.3)	
Age (yr)	62 (46–68)	61.5 (47–69)	52 (46–68)	0.8403	61 (42–67)	62 (48.8–69)	0.0249
*Sex*							
Male (n)	239 (46.0)	61 (38.6)	178 (50.9)	0.01	71 (58.5)	169 (50.1)	0.0757
Female (n)	269 (53.0)	97 (61.4)	172 (49.1)		100 (41.5)	168 (49.9)	0.0757
Height (cm)	169 (162–176)	167 (160–172)	170 (163–176)	0.0037	168 (160–174)	170 (162–176)	0.1423
Weight (kg)	73 (63–84)	67 (58–79)	76 (66–87)	< 0.0001	70.5 (61–82)	74 (64–85)	0.0632
BMI (kg m^−2)^	25.7 (22.5–29.2)	24.2 (20.8–28.3)	26.5 (23.2–29.4)	< 0.0001	25.3 (21.8–29)	26.1 (22.8–29.3)	0.1413
Chronic arterial hypertension	234 (46.1)	70 (44.3)	164 (46.9)	0.59	71 (41.5)	163 (48.4)	0.1438
Pre-induction SBP (mmHg)	140 (125–155)	135 (120–148)	140 (130–155)	< 0.0001	135 (120–150)	140 (130–155)	0.006
Pre-induction MAP (mmHg)	101.7 (92.3–110.0)	96.7 (86.7–106.7)	103.3(101.7–104.9)	< 0.0001	98.3 (88.3–108.3)	101.7 (93.3–110)	0.004
ACEI/ARB	110 (21.7)	37 (23.4)	73 (20.9)	0.5	33 (19.3)	77 (22.8)	0.3590
B-blocker	133 (26.2)	46 (29.1)	87 (24.9)	0.31	42 (24.6)	91 (27)	0.5546
Calcium antagonist	43 (8.5)	15 (9.5)	28 (8.0)	0.57	8 (4.7)	35 (10.4)	0.03
Non-elective procedure	49 (10)	16 (10.1)	33 (9.4)	0.81	14 (8.2)	35 (10.4)	0.43
Regional anesthesia (any)	51 (10)	17 (10.8)	34 (9.7)	0,72	26 (15.2)	25 (7.4)	0.006
*ASA*							
I	47 (9.3)	6 (3.8)	41 (11.7)	0.004	14 (8.2)	33 (9.8)	0.5556
II	246 (48.4)	86 (54.4)	160 (45.7)	0.07	91 (53.2)	155 (46)	0.1241
III	192 (37.8)	55 (34.8)	137 (39.1)	0.35	60 (35.1)	132 (39.2)	0.3704
IV (fisher)	20 (3.9)	8 (5.1)	12 (3.4)	0.46	5 (2.9)	15 (4.5)	0.4770
V (fisher)	3 (0.6)	3 (1.9)	0 (0.0)	0.03	1 (0.6)	2 (0.6)	1.0
CCI (pts)	3 (1–5)	4 (2–5)	3 (1–5)	0.23	3 (1–5)	3 (1–6)	0.2878

The negative outcome was observed in 38 (7.5%) patients, including 32 cases of AKI (6.3%), 3 cases of MI (0.6%) and one event of stroke (0.2%).

Table 3 presents the findings from univariate analyses: patients who met the negative outcome were statistically more often older, underwent longer procedures (≥ 230 min), had episodes of intraoperative hypotension (IOH), were diagnosed with chronic arterial hypertension, were more likely treated with ACEI/ARB or B-blocker, had higher CCI score and higher ASA class.

**Table 3:** Variables related to the presence of the composite outcome (univariate analysis)

Potential hypoperfusive event risk factor	All n = 508 (100.0)	Outcome (−) n = 470 (100.0)	Outcome ( +) n = 38 (100.0)	P-value
Post-induction MAP ≤ 65 mmHg (n)	158 (31.1)	144 (30.6)	14 (36.8)	0.4273
Intraoperative MAP ≤ 65 mmHg (n)	171 (33.7)	149 (31.7)	22 (57.9)	0.0010
Post-induction MAP ≤ 65 mmHg (min)	0 (0–0)	0 (0–0)	0 (0–2.5)	0.2475
Intraoperative MAP ≤ 65 mmHg (min)	0 (0–0)	0 (0–0)	5 (0–10)	0.0014
Post-induction MAP ≤ 65 mmHg only (no intraoperative MAP ≤ 65 mmHg) (n)	77 (15.2)	72 (15.3)	5 (13.2)	0.7211
Intraoperative MAP ≤ 65 mmHg only (no post-induction MAP ≤ 65 mmHg) (n)	109 (21.5)	95 (20.2)	14 (36.8)	0.0164
Both intraoperative MAP ≤ 65 mmHg and post-induction MAP ≤ 65 mmHg (n)	62 (12.2)	54 (11.5)	8 (21.1)	0.0835
Longer procedures ≥ 230 min	249 (49.0)	218 (46.4)	31 (81.6)	0.0001
Age (yr)	62 (46–68)	61 (45–68)	67 (62–74)	0.0002
Sex				0.4738
Male (n)	239 (47.0)	219 (46.6)	20 (52.6)	
Female (n)	269 (53.0)	251 (53.4)	18 (47.4)	
Height (cm)	169 (162–176)	169 (161–176)	168 (164–171)	0.2816
Weight (kg)	73 (63–84)	73.0 (71–74)	73 (64- 83.5)	0.9207
BMI (kg m^−2^)	25.7 (22.5–29.2)	25.6 (25.2–26.2)	27.1 (24.3–29.2)	0.3613
Chronic arterial hypertension	234 (46.1)	205 (43.6)	29 (76.3)	0.0001
Pre-induction SBP (mmHg)	140 (125–155)	140.0 (125–153)	142.5 (130–155)	0,1985
Pre-induction MAP (mmHg)	101.7 (92–110)	101.7 (92–110)	101.5 (95–110)	0.6573
ACEI/ARB	110 (21.7)	97 (20.6)	13 (34.2)	0.0509
B-blocker	133 (26.2)	23 (6.1)	15 (39.5)	0.0529
Calcium antagonist	43 (8.5)	34 (7.3)	4 (9.3)	0.6353
Non-elective procedure	49 (10)	43 (9.1)	6 (15.8)	0.1827
Regional anesthesia (any)	51 (10)	42 (8.9)	9 (23.7)	0.0036
*ASA Class*				
< *III*	290 (57.1)	278 (59.1)	12 (31.6)	0.001
≥ III	218 (42.9)	192 (40.9)	26 (68.4)	
CCI (pts)	3 (3–4)	3 (3–4)	5 (4–6)	< 0,0001

As presented in Table 4, in the multivariate logistic regression model, independent variables significantly associated with outcome were intraoperative MAP ≤ 65 mmHg, longer duration of the procedure ≥ 230 min; chronic arterial hypertension and older age. *P* < 0.01 each.

**Table 4:** Multivariate logistic regression analysis

Potential hypoperfusive event risk factor	B	SE B	Wald χ2	p	OR	95% CI OR
Intraoperative MAP ≤ 65 mmHg	1.13059	0.36949	9.3627	0.0022	3.09	1.50–6.39
Longer procedures ≥ 230 min	1.21613	0.44423	7.4945	0,0062	3.37	1.41–8.06
Chronic arterial hypertension	1.16822	0.45399	6.6216	0.0101	3.21	1.32–7.83
Age	0.034761	0.017353	4.0129	0.0452	1.03	1.00–1.07
Constant	− 6,72,656	1,14,229	34,6761	< 0.0001		
ROC curve	Area under the ROC curve (AUC): 0.808 95% CI 0.770–0.841					

The dependent variable—outcome. 0 = no hypoperfusive event observed and 1 = negative outcome observed

Variables that failed to be included in the regression model: ACI/ARB use, β-blocker use, ASA ≥ III, CCI, Intraoperative MAP ≤ 65 mmHg only (no post-induction MAP ≤ 65 mmHg) (n), Both intraoperative MAP ≤ 65 mmHg and post-induction MAP ≤ 65 mmHg (n), Regional anesthesia

## Discussion

The primary objective of this study was to explore different phases of IOH and verify whether these phases were differently associated with postoperative organ complications. Few studies published to date have focused on the impact of hypotension during the initial phase of anaesthesia—before the onset of surgery—on hypotension that occurs throughout the procedure.

In our homogeneous cohort of patients included in the study, post-induction hypotension (PIH) and intraoperative hypotension (IOH) were found to be two distinct phenomena with different factors associated with their occurrence. PIH was observed in 31% of the study subjects, which is consistent with the percentages reported by other researchers [13]. Female sex, lower body mass and height, lower pre-induction SBP and MAP and higher ASA score were associated with the occurrence of PIH, which is in line with the results reported by other authors [3, 14–16]. However, it should be noted that the results of studies concerning this issue are conflicting. In many cases, the data on the influence of pre-induction blood pressure, gender or drug use on the incidence of PIH are mutually contradictory. This is probably due to the differences in the anaesthesia used, extremely different characteristics of the study populations and the lack of clear definitions of PIH [13].

On the other hand, in our study, the prevalence of IOH was 33.7% in the study population. Our results in this aspect are also consistent with the data of other authors [17]. The association between the occurrence of IOH and variously defined hypoperfusive adverse events has already been reported in multiple studies [3, 4, 18–21]. In our cohort, younger patients (62 vs.61 years old) as well as those treated with calcium antagonists were less prone to develop IOH. This is consistent with the results of our previous analysis showing that older hypertensive patients are less likely to develop IOH, but the effects of hypotension in these patients are more pronounced [5]. Moreover, our analysis showed that lower pre-induction SBP and pre-induction MAP values were associated with a higher incidence of IOH. However, the relevance of pre-induction BP values has often been questioned (in the literature), as have hypotension thresholds defined as a percentage of baseline [20]. It is worth noting that absolute thresholds are more convenient to use in the therapeutic algorithm; preoperative measurements are often ignored and the stress associated with the upcoming procedure has no impact on absolute IOH thresholds during surgery [17]. However, it should be added that according to Futier et al., targeting individualised systolic blood pressure based on preoperative values reduced the risk of postoperative organ dysfunction, as compared to standard management [22].

In our study, MAP ≤ 65 mmHg was used as the threshold for hypotension. This was dictated by the results of our previous analyses in the same group of patients, in which only this determinant was found to be a significant individual risk factor for a negative outcome [5]. The above findings are consistent with the results of other authors [4, 17, 19, 20, 23]. Blood pressure values below the preselected threshold were associated with the increased risk of acute kidney injury, myocardial infarction and stroke—the outcome was present in 38 cases. The relationship between intraoperative hypotension and acute kidney injury seems to be best documented; in our study population, AKI was the most important component of the outcome present in 32 out of 38 cases.

Our key finding is that hypotension occurring during the surgical procedure (IOH), but not after the induction of anaesthesia (PIH), was associated with postoperative complications. IOH, longer procedure time, chronic arterial hypertension and patient age independently predicted postoperative complications. It could be concluded that the difference in the effect of PIH and IOH on the outcome is due to completely different nature of these types of hypotension. The cumulative duration of IOH is much longer, IOH is related to the surgical procedure, injury and shifts in fluid balance. PIH is mainly related to the impairment of physiological reflexes associated with the use of anaesthetics. According to the PQI consensus statement [4] duration and magnitude of SBP below 100 mm Hg and MAP below 60–70 mm Hg during non-cardiac surgery in adults are associated with organ injury. PIH might therefore be harmful to a similar degree as IOH, but IOH due to its characteristics can last longer, making it important in determining the outcome.

In a large cohort analysis published by Maheshwari et al., 36% of the recorded hypotension events defined as MAP < 65 mmHg occurred during the post-induction phase of the procedure; the relationship was found between the presence of PIH and the prevalence of AKI within 7 post-operative days. Another study suggesting the negative impact of PIH on the postoperative outcome (increased ICU length of stay, mortality and number of postoperative complications) is the work of Green and Butler [24]. In our study the recorded PIH time accounted for only 22.8% of the total hypotension time and was not related to the occurrence of the negative outcome. The results of our study and the study discussed above, however, should be compared with caution due to the differences in sample size, study design and heterogeneity of the patient group. The IOH time accounted for 5.17% of the total intraoperative time, while PIH time accounted for 8.91% of the cumulative time between anaesthesia induction and skin incision. However, it should be noted that in our cohort of patients, where the procedures were relatively long, the time before the incision represented only 17.23% of the cumulated procedure time (Fig. 2). The prevalence of PIH varies significantly between different studies mostly because of the lack of unified criteria of PIH. Ida et al. have reported the incidence of PIH defined as the relative SBP drop of at least of 25% at the level of 96,8% in the period from tracheal intubation to the onset of surgery [25]. Green and Butler have reported the occurrence of hypotension within 15 min after tracheal intubation in 60% of subjects undergoing blood vessel surgery. They have defined hypotension as SBP lower than 80 mmHg, a decrease in SBP of more than 20% compared to baseline values, or a decrease in MAP below 60 mmHg [8]. At the other extreme are the papers by Reich et al. and Sudfeld et al. reporting PIH prevalence of 9 and 18%, respectively [3, 14]. The often-discussed effect of general anaesthetic drugs may be completely neglected in the case of our study, as all patients in our cohort received standard propofol and fentanyl for anaesthesia induction. Since hypotension in the period after induction of anaesthesia is a relatively short episode in relation to the duration of the entire procedure and is often a side effect of the use of anaesthetic drugs whose effect subsides with the onset of surgery, the real impact of PIH on perioperative complications seems difficult to estimate and should be estimated individually for each clinical situation. Presence and effect of PIH is largely determined by the patient's baseline risk factors and the anaesthetic management. It is not insignificant that blood pressure during and immediately after the induction of anaesthesia can be controlled to a large extent by anaesthetists. Strategies described include the administration of prophylactic fluid boluses (colloids and crystalloids), pre-emptive administration of ephedrine or phenylephrine before or during the induction of anaesthesia and reducing the dose of volatile anaesthetics.[26–30].

The 2022 ESA and ESC guidelines emphasise the importance of adequate haemodynamic monitoring in patients undergoing non-cardiac surgery; in surgical patients at increased risk of cardiovascular events, blood pressure should be monitored invasively on a continuous basis. Non-invasive monitoring remains the standard for low-risk patients undergoing low- and moderate-risk surgery. In the guidelines, the authors also describe the confusing and often impossible to set strict thresholds for different types of hypotension, emphasising the need to personalise the approach to patients [9]. To date, hypotension is treated reactively after low blood pressure values have already occurred. Studies aiming to identify patients at risk of PIH based on ultrasound measurements (e.g. of the jugular veins) or on Machine Learning algorithms seem interesting [15, 16, 31].

By identifying potential risk factors for the development of PIH, IOH and the occurrence of composite outcome, our study fits into the broader discussion on personalising the approach to surgical patients. Another strength of our study is the homogeneity of the population—all patients received general anaesthesia, underwent gastrointestinal surgery, and in most cases were administered the same drugs (anaesthesia was induced with propofol in 94.5% cases and maintained with sevoflurane in 87.6% cases). This could reduce the bias resulting from specific procedural conditions. As far as limitations are concerned, the generalisability of our study might be limited due to a relatively small sample size of our population. The size of our cohort may be underpowered to detect certain associations. Moreover, this is an observational study and therefore can only address association and not causality. Thirdly, MAP was calculated using a formula, not measured directly during the procedure. There might be a difference between MAP values derived from an equation and those obtained from oscillometric BP measuring. Additionally, BP was recorded at 5-min intervals—the real, exact time of intraoperative hypotension could be mismatched. Arterial pressure measurements were performed on one arm only due to technical reasons; therefore, there is the possibility of an observational error, which could be eliminated by performing parallel measurements on both arms. Moreover, we did not assess the BP value in relation to the perioperative fluid balance, which might have affected our results. Another issue is associated with outcome reporting. There is a risk that some asymptomatic hypoperfusion events were not reported due to suboptimal monitoring and insufficient diagnostics after discharge. In our study, blood pressures after discharging the patient from the operating theatre were not studied. However, the role of the postoperative care period in the adverse outcomes observed cannot be underestimated.

## Conclusions

In patients undergoing abdominal surgery, the presence of IOH defined as MAP ≤ 65 mmHg is relevant to postoperative organ complications, while the presence of PIH does not appear to be of such significance. Because cumulative duration of PIH and IOH differs significantly, especially in long-lasting procedures, direct comparison of the influence of PIH and IOH on outcome separately may be biased and should be taken into account in data interpretation. PIH might therefore be harmful to a similar degree as IOH, but IOH due to its characteristics can last longer, making it important in determining the outcome. Further research is needed to deeply investigate this phenomenon. Longer procedure time, chronic arterial hypertension, and age independently predicted postoperative complications. Utilising the identified risk factors of PIH, IOH and hypoperfusive outcome and their skilful avoidance may allow for prevention of hypotension or implementation of its treatment at the appropriate time point.

## Data Availability

The data that support the findings of this study are available on request from the corresponding author - S.Czajka.
